# Secretory Carrier Membrane Protein (SCAMP) deficiency influences behavior of adult flies

**DOI:** 10.3389/fcell.2014.00064

**Published:** 2014-11-18

**Authors:** JiaLin C. Zheng, Chook Teng Tham, Kathleen Keatings, Steven Fan, Angela Yen-Chun Liou, Yuka Numata, Douglas Allan, Masayuki Numata

**Affiliations:** ^1^Department of Biochemistry and Molecular Biology, University of British ColumbiaVancouver, BC, Canada; ^2^Department of Cellular and Physiological Sciences, University of British ColumbiaVancouver, BC, Canada

**Keywords:** *Drosophila*, mutant, vesicular trafficking, climbing assays, lifespan, mobility, odor-associated learning, long-term memory

## Abstract

Secretory Carrier Membrane Proteins (SCAMPs) are a group of tetraspanning integral membrane proteins evolutionarily conserved from insects to mammals and plants. Mammalian genomes contain five SCAMP genes SCAMP1-SCAMP5 that regulate membrane dynamics, most prominently membrane-depolarization and Ca^2+^-induced regulated secretion, a key mechanism for neuronal and neuroendocrine signaling. However, the biological role of SCAMPs has remained poorly understood primarily owing to the lack of appropriate model organisms and behavior assays. Here we generate *Drosophila Scamp* null mutants and show that they exhibit reduced lifespan and behavioral abnormalities including impaired climbing, deficiency in odor associated long-term memory, and a susceptibility to heat-induced seizures. Neuron-specific restoration of *Drosophila Scamp* rescues all *Scamp* null behavioral phenotypes, indicating that the phenotypes are due to loss of neuronal *Scamp*. Remarkably, neuronal expression of human SCAMP genes rescues selected behavioral phenotypes of the mutants, suggesting the conserved function of SCAMPs across species. The newly developed *Drosophila* mutants present the first evidence that genetic depletion of SCAMP at the organismal level leads to varied behavioral abnormalities, and the obtained results indicate the importance of membrane dynamics in neuronal functions *in vivo*.

## Introduction

Secretory Carrier Membrane Proteins (SCAMPs) are evolutionarily conserved tetra-spanning integral membrane proteins that are broadly associated with organellar and plasma membranes (Law et al., 2012). The *Drosophila* genome contains a single *Scamp* gene on the X chromosome whereas mammals have five genes, SCAMP1-SCAMP5. Neurotransmission involves membrane-depolarization and Ca^2+^-dependent regulated secretion of synaptic vesicles or dense core vesicles (DCVs) targeted to pre-synaptic membrane, followed by docking and fusion in order to release neurotransmitters (Burgess and Kelly, 1987; Kim et al., 2006). Earlier biochemical studies suggested crucial roles of SCAMPs in these processes (Guo et al., 2002; Liu et al., 2005; Liao et al., 2008). Isolation of varied neuron-enriched membrane-bound transporters as SCAMP-binding proteins and characterization of SCAMPs' role as targeting regulators have provided additional insights. For instance, SCAMPs bind to neurotransmitter transporters (solute carrier 6, SLC6) and regulate their cell-surface targeting (Muller et al., 2006; Fjorback et al., 2011). In light of the significant role of cell-surface SLC6 in emotion and social behavior (Chen et al., 2004; Risch et al., 2009; Kristensen et al., 2011), potential impact of SCAMPs-mediated targeting of these transporters is substantial. Another potentially important SCAMP-binding partner is the neuron-enriched pH-regulator Na^+^/H^+^ exchanger NHE5. NHE5 is predominantly associated with endocytic recycling organelles in resting cells (Diering et al., 2013), and SCAMPs play an important part in targeting NHE5 from endosomes to the plasma membrane in a non-neuronal heterologous expression system (Diering et al., 2009). In mature neurons, neuronal activation acutely recruits NHE5 to dendritic spines, regulates pH therein and greatly influences dendritic spine formation and remodeling (Diering et al., 2011). Thus, regulated targeting of NHE5 may modulate synaptic plasticity. Although the involvement of SCAMPs in activity-dependent dendritic targeting has not been tested, SCAMPs' proposed role in Ca^2+^- and depolarization-induced membrane fusion and docking makes this possibility intriguing (Diering and Numata, 2014). Another class of organelle-membrane-bound (Na^+^, K^+^)/H^+^ exchange NHE7 was also identified as a SCAMP-binding protein (Lin et al., 2005). Curiously, a multivariate association study using brain-imaging data and genome-wide single nucleotide polymorphisms identified NHE7 as a novel candidate gene for late-onset Alzheimer's disease (Meda et al., 2012). In regard to the disease-association, positional cloning has identified SCAMP5 as a candidate for autism susceptible gene (Castermans et al., 2010). Moreover, DNA microarray analysis identified SCAMP1 expression is decreased in the prefrontal cortex of schizophrenia patients (Arion et al., 2007), which may be associated with the symptomatic activity of this disease (Le-Niculescu et al., 2009).

The possible involvement of SCAMPs in both regulated-secretion and membrane targeting makes these evolutionarily conserved tetra-spanning integral membrane proteins promising candidates for neuronal regulators. Despite the previous biochemical and cell biological results suggesting the fundamental significance of SCAMPs in neurons, biological roles of SCAMPs in live organisms remain untested due to the lack of suitable model organisms. We have now generated a null allele to *Drosophila Scamp* by imprecise excision of a P-element and report pronounced behavioral abnormalities in adults: an accelerated age-dependent decline in climbing ability, defective learning and long-term memory retention, and heat-induced seizure susceptibility.

## Materials and methods

### Reagents

Biochemical reagents were of analytical grade or better and were obtained from Sigma-Aldrich (St Louis, MO, USA), Fisher Scientific Company (Ottawa, ON, Canada) or Bioshop Canada Inc. (Burlington, ON, Canada) unless otherwise indicated. Oligonucleotides were obtained from Invitrogen (Burlington, ON, Canada). Rabbit polyclonal antibodies against the synthetic peptide MSGSGLDENPFGEPNLDN(C-amide) corresponding to the N-terminal 18 amino acids for *Drosophila SCAMP* were raised by YenZym Antibodies LLC (South San Francisco, CA, USA). Anti-myc-epitope mouse monoclonal 9E10 and rabbit polyclonal A14 antibodies that recognize EQKLISEEDL were purchased from Santa Cruz Biotechnologies, Inc. (Santa Cruz, CA, USA), anti-β-tubulin and anti-nervana antibodies were obtained from Developmental Studies Hybridoma Bank (Iowa City, IA, USA). Horseradish Peroxidase (HRP)-conjugated goat anti-mouse and anti-rabbit antibodies were purchased from Jackson Immuno Research Laboratories, Inc. (West Grove, PA, USA).

### *Drosophila* strains

Strains used were: *y*1*w*67*c*23P{*w*[+*mC*]=*GSV1*}*GS*1041/*Binsinscy* (DGRC number 200072, Drosophila Genetic Resource Center; Kyoto, Japan), isogenized *w*^1118^ (*iso*-*w*^−^), *elav-GAL4*^*C1*55^, *w*^−^;*Sp/CyO*;Δ2-3; *Sb/TM*6(±Δ2-3) and y[^1^]w[*]P{UAS-mCD8::GFP.L}*Ptp4E*^*LL*4^ *Smox*^*MB*388^ *P{neoFRT}19A/FM7c* (Stock number 44384, Bloomington Drosophila Stock Center; Bloomington, IN, USA).

### Generation of *Scamp* deficiency mutants

The P-element was mobilized from the *y1w67c23P{w[+mC]=GSV1}GS1041* strain (Drosophila Genetic Resource Center) by crossing to Δ2-3 transposase. Approximately 300 mosaic-eyed progeny were crossed with the *FM7c* balancer y[^1^]w[*]P{UAS-mCD8::GFP.L}Ptp4E^LL4^ Smox^MB388^ P{neoFRT}19A/FM7c (Bloomington Drosophila Stock Center) for two generations and the white eyed balanced progeny were PCR screened using the primers listed in Table 1. The molecular lesion generated by imprecise excision was determined by direct sequencing of genomic DNA from homozygous flies. For transgenic rescue experiments, *Scamp*^63A^, *elav-GAL4*^*c*155^ strain was generated.

**Table 1:** **Primers used for *Drosophila Scamp* null mutant-screening**.

**Primer**	**Sequence**	**Genomic position**
For 1	5′- ACA CTA GAA TTC CAA TTG CGG AAT G-3′	15450286–15450262
For 2	5′- GGT CCT AAA AAA TCA GAC CAT TGT ATA CCA G-3′	15450091–15450061
Rev 1	5′-GAC GGC ATT CTA GGA ATT GTT TAT GC-3′	15447149–15447174
Rev 2	5′-CAC GTT GGC CAA CAA CGT CAT GGT G-3′	15447996–15448020
Rev 3	5′-TTG TAA TCC TCA AGA GAG ACA AGG G-3′	15449441–15449465

### Generation of transgenic flies

A cDNA encoding *Drosophila Scamp* was isolated by RT-PCR using a *Drosophila* embryo mRNA (Clontech, Mountain View, CA, USA) as a template. Human SCAMP1 and SCAMP5 cDNAs were previously isolated in our laboratory as described (Lin et al., 2005). In brief, first strand cDNA synthesized from human brain total RNA by the use of random primers for reverse transcription was used as a template for PCR. N-terminal myc-tag was introduced by PCR using the primers summarized in Table 2 and ligated into the *pUASTattB* vector (Groth et al., 2004; Fish et al., 2007; Markstein et al., 2008). The sequence of the N-terminally myc-tagged clones was verified and transgenes were site specifically integrated into the *attP2* genomic site by *phiC31* recombinase by BestGene Inc. (Chino Hills, CA, USA).

**Table 2:** **Primers used for cloning of *Drosophila Scamp*, human *SCAMP1* and human *SCAMP5***.

**Primer**	**Sequence**
Myc-DmSc For	5′-CGG GAT CCT CGA GAA AAA *ATG GAG CAG AAG CTG ATC TCC GAG GA*G GAC CTG TCC GGC TCC GGT CTC GAC GAG-3′
DmSc Rev	5′-GCT CTA GAG TAC CTG CTG TTG TTA AAC TGC-3′
Myc-hSc1 For	5′-CGG GGT ACC AAA AA*A TGG AGC AGA AGC TGA TCT CCG AGG AGG* ACC TGT CGG ATT TCG ACA GTA ACC C-3′
hSc1 Rev	5′-CGG GGT ACC TTA AAT CTG GTT ACC CTT GAA AGC ATT CTG-3′
Myc-hSc5 For	5′-CGG ATC CCT CGA GAA AAA *ATG GAG CAG AAG CTG ATC TCC GAG GAG GAC CTG* GCA GAG AAA GTG AAC AAC TTC CC-3′
hSc5 Rev	5′-GCT CTA GAT TAC ATC TCA TTG GAG TAC GTG TAA TTG GG-3′

*Restriction sites were underlined and the sequence for myc-tag was italicized*.

### Western blot

Adult flies were quickly frozen in liquid nitrogen and the removed heads were lysed in RIPA buffer containing 140 mM NaCl, 20 mM Tris-HCl pH 8.0, 1% NP40, 0.1% SDS and 0.5% sodium deoxycholic acid freshly supplemented with proteinase inhibitor cocktails (Roche Diagnostics, Laval, PQ, Canada). The lysate was cleared by centrifugation at 16,000 *r.c.f*. for 15 min at 4°C twice, and the protein concentration of the supernatant was determined with the Bradford reagent (BioRad, Hercules, CA, USA). The denatured sample was resolved in a 12% SDS-PAGE gel and transferred to a 0.45 μm pore size polyvinylidene fluoride (PVDF) membrane (EMD Millipore, Billerica, MA, USA). The blot was blocked in 5% skim milk in PBS-T (0.075% Tween 20 in PBS pH 7.4) and probed with anti-*Drosophila* SCAMP, anti-myc, or anti-nervana antibody at room temperature for 1 h. After extensive washing with PBS-T, the blot was incubated with HRP-conjugated secondary antibody. Following another washing in PBS-T, signal was detected with Luminata Forte Western chemilluminescent horseradish peroxidase substrate (Millipore, Billerica, MA, USA).

### Fly stock maintenance for behavior experiments

Homozygous *Scamp* deficiency mutant female flies were outcrossed with *iso-w^−^* male, and the male *Scamp* hemizygous F1 progeny was tested for behavior, unless otherwise stated. Two independently-generated *Scamp* deficiencies were crossed and the heteroallelic *Scamp* deficient female F1 progeny was also tested for certain behaviors. Fly stocks were maintained at 22°C or 25°C and 70% relative humidity with 12 h/12 h light/dark cycles on standard cornmeal-yeast-based food. Flies to be tested in behavior assays were collected from freshly eclosed stocks, and transferred to fresh food vials prior to the experiment. All the experiments were done in the early to midafternoon in order to avoid variation due to circadian rhythm.

### Lifespan analysis

Null *Scamp*^63A^ and precise excision *Scamp*^211A^ homozygotes were collected within 16 h after eclosion and raised at 25°C in a moisture- and light-controlled environmental room. Surviving flies were counted every second day. Kaplan-Meier survival curves were generated by calculating the ratio of live flies and plotting the value as a function of incubation time.

### Climbing assays

Climbing assays were performed as described previously (Leal and Neckameyer, 2002; Martinez et al., 2007; Perkins et al., 2010) with some modifications. Age-matched adult flies of each genotype were transferred without anesthesia to an odorless, clean polystyrene climbing-test-tube and kept in the dark for approximately 10 min prior to the experiment. To assay climbing, the test chamber was tapped three times to bring all flies down to the bottom; we counted the number of flies that climbed past the 7 cm line, from the bottom, within 8 s. During climbing, flies were illuminated only from above to facilitate phototactic climbing. Mean numbers of flies crossing the 7 cm line was determined in 4 or 5× replicates per assay, and we performed 5 to 9 assays per genotype. All experiments were recorded with video for data collection.

### Olfactory conditioning learning and memory assay

House-made T-maze equipment and aversive odor associated learning assays were built and conducted as previously described (Tully and Quinn, 1985), with slight modifications. First, we entrained flies of all genotypes for odor-associated long-term memory. Approximately 130 post-eclosion flies (3–5 days old) of each genotype were transferred to a light-protected training chamber without anesthesia and exposed to a constant flow of odorless air (OLA) for 30 min. Thereafter, the following entrainment procedure was followed: (i) exposure to methylcyclohexanol (MCH) while being mildly electrically shocked with 0.5 s 60 V pulses (approximately 0.75 mA) given at 1.5 s intervals for 60 s, (ii) 45 s of OLA, (iii) 60 s of 3-octanol (OCT), and (iv) 45 s of OLA. After a 15 min spacing period, the entrainment cycle was repeated 10 times, each followed by a 15 min spacer. To test odor-associated long-term memory, trained flies were maintained at 22°C for 20 h before testing. Trained flies were transferred to the T maze apparatus without anesthesia, and exposed to MCH (shock-associated odor) and OCT (shock-unassociated odor) injected from opposite ends of the apparatus, but converging at the T maze choice point. Flies were forced to move from the choice point toward either MCH or OCT over a 2.5 min period by constant gentle agitation of the sliding center compartment at the choice point. At the end of the assay, the sliding center compartment was pulled up to separate flies on either end, and the number of flies on each side was counted. Experiments were carried out in the dark to minimize any phototaxis. Similar experiments using OCT as an associated odor and MCH as an non-associated odor were conducted and the learning index (LI) was calculated (Tully and Quinn, 1985). LI= (the fraction of flies avoiding the shock-associated odor) − (the fraction of flies avoiding the shock-unassociated odor). Reciprocal experiments were conducted using OCT as a shock-associated odor and MCH as a shock-unassociated odor, and the average value was defined as LI.

### Heat-induced seizure assays

Heat-induced seizure assays were carried out as described previously (Hoeffer et al., 2003) with some modifications. Newly eclosed adult flies of each genotype were grouped in triplicate in regular fly food vials (10–12 flies per vials), and were grown for 4 days at 22°C prior to the experiments. When the *UAS-GAL4* system was used, flies were incubated at 29°C for 3 days followed by 1-day incubation at 22°C. For seizure induction, flies were transferred to fresh polystyrene fly vials and immersed in a 42.3°C water bath for 3 min at which time all the flies were paralyzed. The fly vials were then transferred to 22°C and the number of standing flies that recovered from the heat-shock was counted every minute for up to 30 min. Experiments were repeated 7 times using different flies and all data were combined in the presented graphs of Kaplan-Meier failure function [1-S(t + 0)].

### Statistics

Statistical analyses were conducted using Stata/IC 10.1 (StataCorp LP, College Station, TX, USA). Log-rank tests were used for *Drosophila* survival analysis and for the analysis of recovery time of heat-induced seizure assays. Means of two groups were compared with Student's *t*-tests or Wilcoxon rank-sum tests. All hypothesis tests are two-sided. Repeated measures ANOVA was used to analyze the longitudinal climbing assay data, with age as a within-subject factor, taking the proportion of climbed flies in each vial as the unit of analysis.

## Results

### Generation of *Scamp* null mutant flies

**Figure 1:**
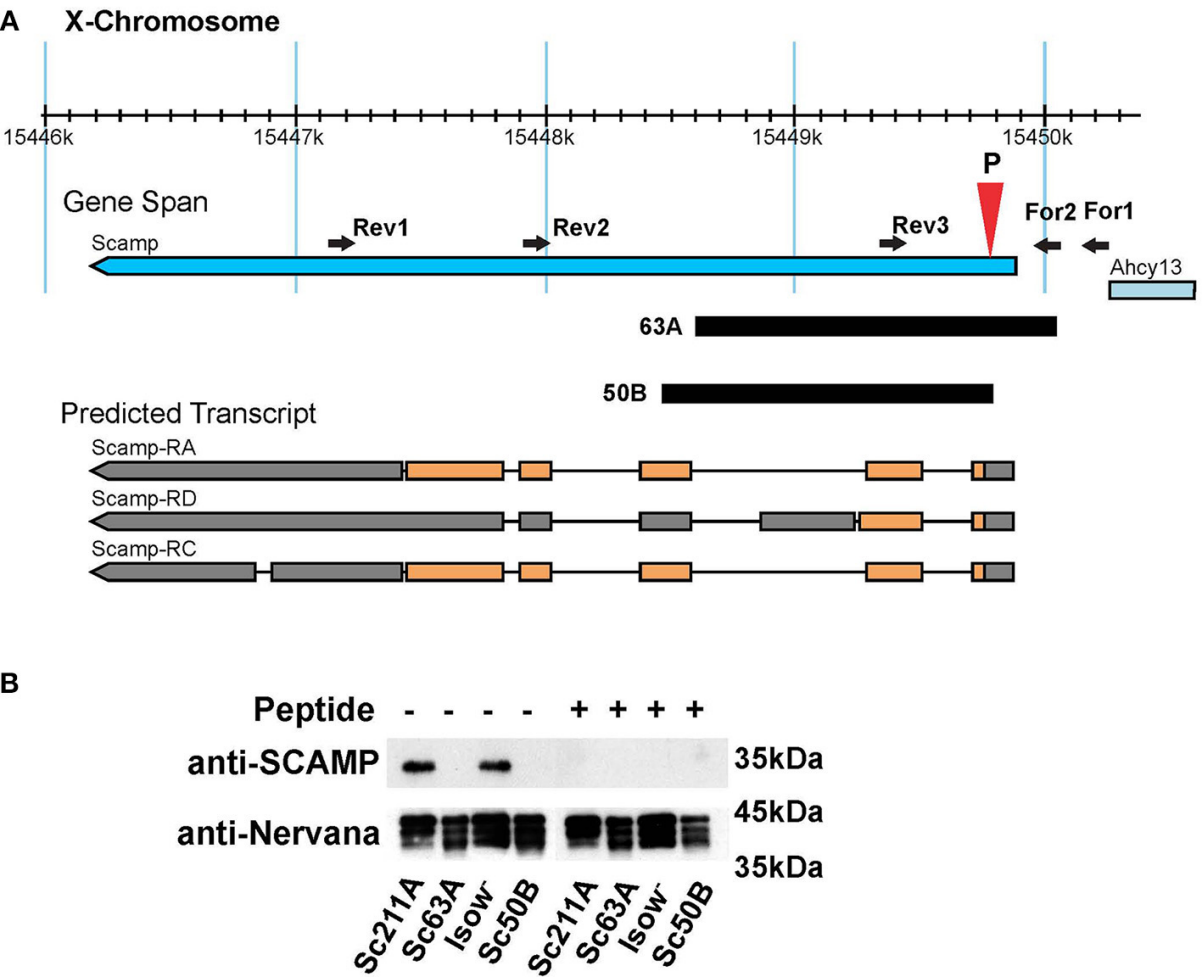
**Generation of *Scamp*-deficiency mutants. (A)** A schematic of the genomic region of the *Scamp* gene and the deficiency mutants used in the study. The position of the deletion (63A and 50B) is indicated by black blocks; PCR primers (forward primers For1 and For2, and reverse primers Rev1, Rev2, and Rev3) used for screening mutants are indicated as arrowheads. P shows the position of *P{GSV1}GS1041* insertion. Orange blocks, coding regions; Gray blocks, untranslated exons; Black lines, introns. Arrows indicate the direction of transcription. Although western blot reveals a single band with the expected size from RA and RC, the possible existence of a truncated form translated from RD cannot be completely excluded. Figure modified from Flybase (http://flybase.org/). **(B**) SCAMP expression in homozygous precise and imprecise excision mutants was tested by western blot. Pre-incubation of the SCAMP antibody with the epitope peptide diminished the signal to undetectable levels, assuring specificity of the antibody. The blot was probed with anti-Nervana antibody as a loading control.

By mobilizing the *P{GSV1}GS1041*, several imprecise excision mutants were isolated (Figure 1A). In the current study, *Scamp*^63A^ was used for most of the behavioral analysis whereas *Scamp*^50B^ was used to generate heterozygous *Scamp*^63A^/*Scamp*^50B^ null alleles to characterize female behaviors. As controls, precise excision revertant lines, *Scamp*^211A^ and *Scamp*^209A^ were used. To test protein expression in these mutants, a polyclonal antibody against the N-terminal 18 amino acids of *Drosophila* SCAMP was raised in rabbit. A single band approximately 35 kD in size was detected by western blot in lysates isolated from *Scamp*^211A^ and *iso-w* (Figure 1B). Pre-incubation of the primary antibody with the epitope peptide eliminated this band. No appreciable signal was detected in *Scamp*^63A^ and *Scamp*^50B^, demonstrating the antibody's specificity. Homozygous deletion mutant flies are viable, fertile and have no apparent morphological abnormalities.

### *Scamp* null adult flies have shortened lifespan

**Figure 2:**
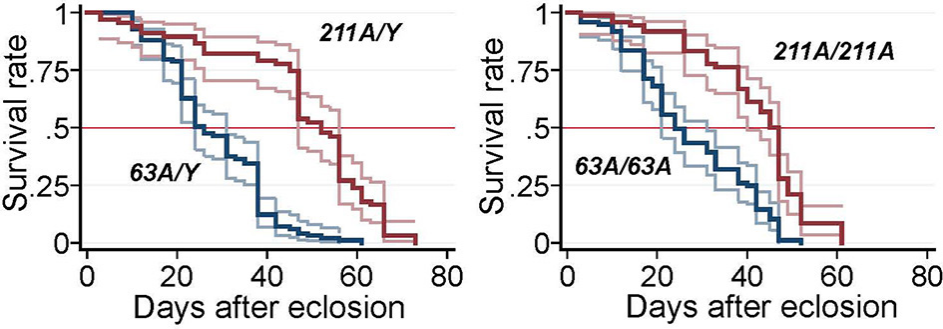
**Survival curves of *Scamp* mutant flies**. Null males (*Sc63A/Y*, *N* = 99) and precise excision controls (*Sc211A/Y*, *N* = 67), null females Sc63A/63A (*N* = 97) and controls *Sc211A/Sc211A* (*N* = 72) were raised at 25°C and the number of survivors was counted every other day. Pointwise 95% confidence bands of the estimated survival functions are shown by outer lines in pale colors.

Decreased lifespan is frequently associated with neurodegenerative disorders in *Drosophila* and is often used as a “straightforward first look” at the phenotype (Lessing and Bonini, 2009). We therefore tested the survival of adult flies. The median lifespan for revertant *Scamp*^211A^ males was 52 days whereas that of *Scamp*^63A^ males was substantially reduced to 26 days (−40.9%) (*p* < 0.001, log-rank test) (Figure 2). A similar reduction of life span was observed in *Scamp*^63A^ homozygous females (34.1% reduction compared to controls, log-rank test *p* < 0.001).

### *Scamp* deficient adult flies exhibit slower climbing behavior

**Figure 3:**
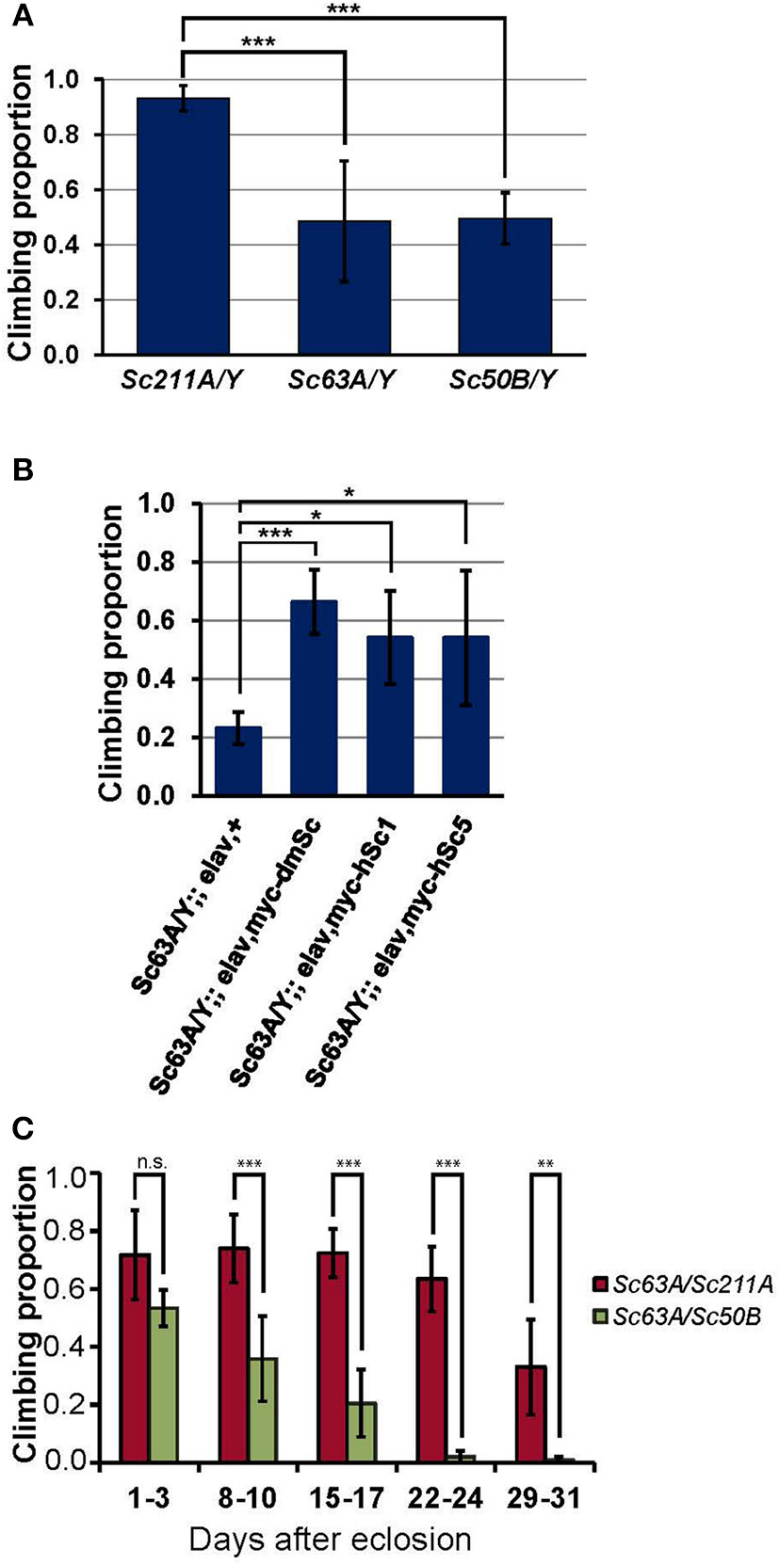
***Scamp* nulls exhibit climbing impairment. (A)** Outcrossed null males (*Sc63A*/*Y* and *Sc50B*/*Y*) 5–8 days post-eclosion showed significantly retarded climbing than age-matched *Sc211A*/*Y* controls. Results of climbing tests are shown as averages of *N* = 6, *N* = 9, *N* = 6 different sets of flies for *Sc211A*/*Y, Sc63A*/*Y, Sc50B*/*Y*, respectively. **(B)** Pan-neuronal expression of the myc-tagged *Drosophila Scamp*, human *SCAMP1* or *SCAMP5* rescued behavior defects of *Scamp*-deficiency. *N* = 4 sets per genotype within 17–20 days of eclosion were assayed. **(C)** Heteroallelic null females (*Sc63A*/*Sc50B*) climbed markedly slower than heterozygous females (*Sc63A*/*Sc211A*), suggesting the recessive effect of *Scamp*-deficiency. The retarded climbing of nulls became apparent after 8–10 days of eclosion. *N* = 5 sets per genotype (housed *N* = 9–14 flies per vial) were tested weekly for 4 weeks. Mean ±*SD*; ^***^p < 0.001; ^**^p < 0.01; ^*^p < 0.05 by Student's *t*-test.

Impaired climbing behavior is frequently associated with neurological disorders, and climbing is one of the most commonly assayed behaviors because of the sensitive and robust nature of the assay that allows for testing a large number of flies (Lessing and Bonini, 2009). Homozygous *Scamp* deficient females (*Scamp*^63A^ and *Scamp*^50B^) and homozygous revertant *Scamp*^211A^ females were crossed with isogenized (*isow^−^*) males, and the F1 male nulls (*Scamp*^63A^/*Y* and *Scamp*^50B^/*Y*) and *Scamp*^211A^/*Y* controls were subjected to climbing assays 5–8 days after eclosion. Climbing of *Scamp*^63A^/*Y* and *Scamp*^50B^/*Y* was significantly slower than the *Scamp*^211A^/*Y* (*p* < 0.001 by Student's *t*-test, Figure 3A) where the two null mutants exhibited almost identical degrees of impairment. We next asked whether transgenic expression of *Drosophila Scamp*, human SCAMP1 and SCAMP5 can rescue the climbing phenotype of *Drosophila Scamp* nulls. When pan-neuronally expressed by *elav-GAL4*^*c*155^, all the heterologous human and *Drosophila* proteins were expressed similarly (Supplementary Figure 1). Transgenic expression of *Drosophola Scamp* under pan-neuronal promoter almost fully rescued the *Scamp* null climbing phenotype (Figure 3B). Either human SCAMP1 or SCAMP5 in *Scamp* null mutants substantially ameliorated the climbing impairment (*p* < 0.05 by Student's *t*-test), suggesting the conserved role of SCAMPs in climbing behavior. The neuronal expression of any SCAMP gene in heterozygous *Scamp*^63A^/+ females did neither deteriorate nor enhance the climbing behavior, further supporting the specificity of rescue experiments (data not shown). We next examined whether female *Scamp*^63A^/*Scamp*^50B^ heteroallelic nulls at different ages also elicit retarded climbing. *Scamp* nulls showed significantly slower climbing than *Scamp*^63A^/*Scamp*^50B^ heteroallelic mutants at all ages except for 1–3 days post-eclosion (*p* < 0.001 or *p* < 0.01 by Student *t*-tests, Figure 3C). Climbing ability of heterozygous *Scamp*^63A^/*Scamp*^211A^ flies started to show only a slight decline (−11% on average) at 22–24 days after eclosion whereas *Scamp* nulls (*Scamp*^63A^/*Scamp*^50B^) exhibited a significant decline (−24% compared to the initial proportion) as early as 8–10 days post-eclosion. Results from the repeated measures ANOVA supported that *Scamp* nulls perform worse and the degree of age-dependent decline significantly differs between the two strains [the interaction term of age and strain was statistically significant with *F*_(4, 354)_ = 15.85, *p* < 0.001], indicating that *Scamp* deficiency accelerates age-dependent climbing impairment.

### *Scamp* is necessary for olfactory-associated memory formation

**Figure 4:**
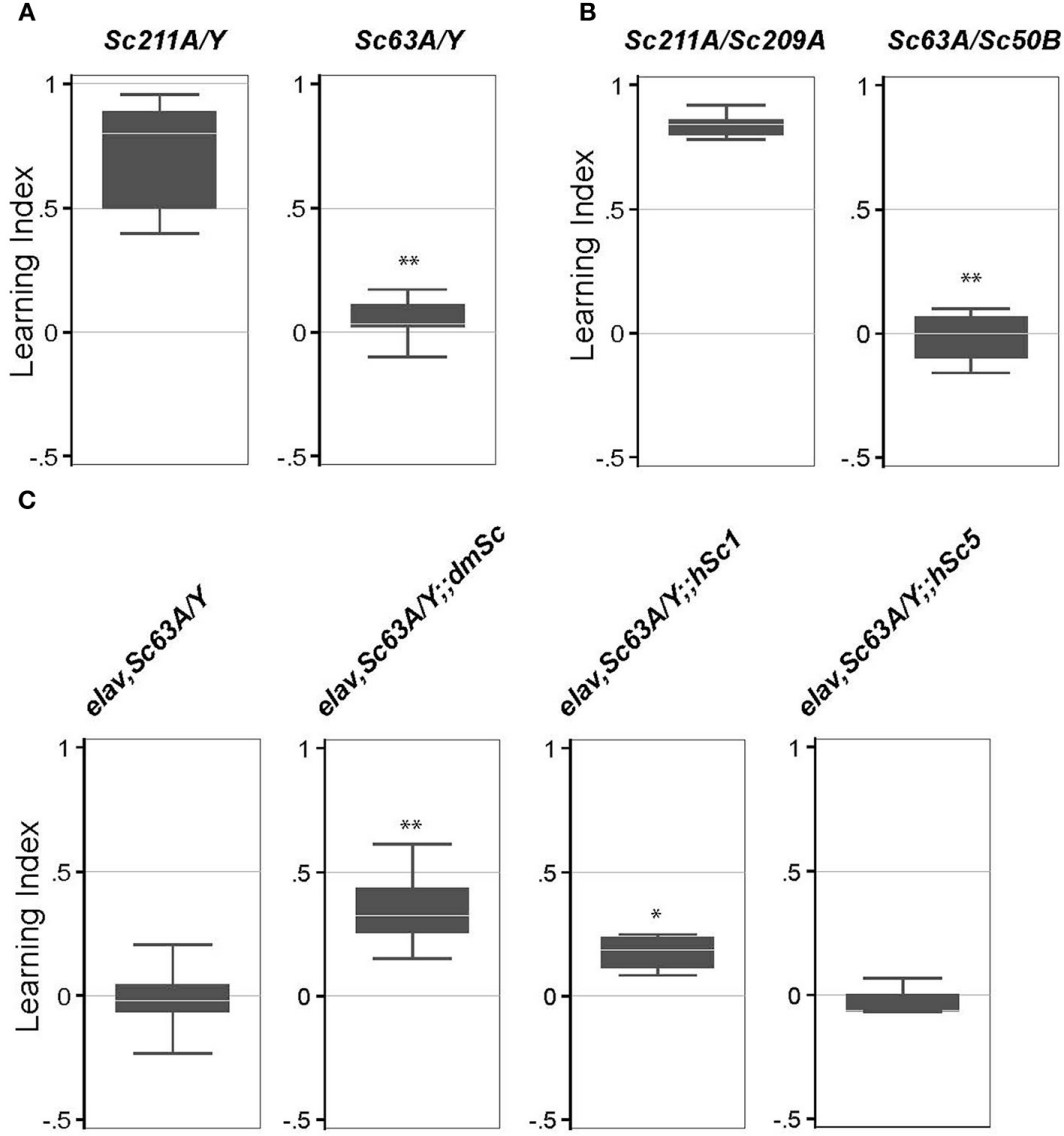
**Long-term memory is impaired in *Scamp* null mutants. (A)** Long-term memory of the outcrossed male progeny (*Sc211A/Y* and *Sc63A/Y*) was tested 3–5 days post-eclosion. *N* = 6 experiments were conducted as shown in the box plot. **(B)** Long-term memory of female heterozygous *Scamp* nulls (*Sc63A/Sc50B*) and precise excision controls (*Sc211A/Sc209A*) was tested 3–5 days post-eclosion. *N* = 6 for *Sc211A/Sc209A* and *N* = 4 for *Sc63A/Sc50B*. **(C)** *elav-GAL4* and *Scamp*^63A^ was genetically combined with *UAS-myc-dmSc, myc-hSc1* or *myc-hSc5* and the long-term memory of the age-matched male progeny was assessed. *N* = 4–6 assessments per strain were conducted. ^**^p < 0.01; ^*^p < 0.05 by Wilcoxon rank-sum test.

A previous study showed that *trans*-heterozygosity for the long-term memory defective mutant CG32594 (*ben*) and *Scamp* (a P-element insertional mutant) led to a defect in long-term memory (Zhao et al., 2009). However, the effects of a *Scamp* null on long-term memory formation are unknown. To address this, we used an established aversive olfactory associated learning assay that uses electric shock for reinforcement. When exposed to 4-methylcyclohexanol (MCH) or 3-octanol (OCT) in the T-maze test tube under the optimized experimental condition, all genotypes evaded the odor source and moved into the opposite chamber within 2.5 min, suggesting that *Scamp* mutants sense odors and their odor-avoidance movement is intact. *Scamp* null adults and age-matched revertant controls were subjected to 10 cycles of training sessions consisting of an exposure with a shock-associated- and unassociated-odors. Both genotypes reacted similarly to electric shock by freezing, falling and jumping. This memory persisted in male revertant flies after 24 h, as they showed a strong tendency to avoid the shock-associated odor 24 h after training [Learning Index (*LI*) = 0.72 ± 0.22 (mean ± SD) Figure 4A]. In contrast, *Scamp* nulls displayed an almost even distribution between the shock-associated and unassociated odors, showing a significant difference in LI (*LI* = 0.046 ± 0.092, *p* < 0.01 by Wilcoxon rank-sum test). Similarly, heteroallelic null females exhibited significant impairment in long-term memory, as opposed to female homozygous revertants (*LI* = 0.015 ± 0.11 vs. 0.84 ± 0.049, *p* < 0.01 by Wilcoxon rank-sum test, Figure 4B). These results indicate that *Scamp* functions in learning and long-term memory in both males and females. We next tested the effect of neuronal restoration of *Drosophila Scamp*, human *SCAMP1* or *SCAMP5* on the long-term memory deficit of *Scamp* null males. Inclusion of the *Drosophila UAS-Scamp* (*dmSc*) significantly improved the Learning Index (LI), compared to the *Scamp* nulls that had *elav-GAL*^*C*155^ (*LI* = 0.35 ± 0.16 vs. −0.016 ± 0.14, *p* < 0.01 by Wilcoxon rank-sum test, Figure 4C). Interestingly, transgenic expression of human SCAMP1 (*hSc1*) but not SCAMP5 (*hSc5*) also restored the LI of *Scamp* nulls albeit to a lesser extent, suggesting some conserved roles between humans SCAMP1 and *Drosophila SCAMP* in long-term memory formation.

### *Scamp* null mutant flies exhibit susceptibility to heat-induced seizures

**Figure 5:**
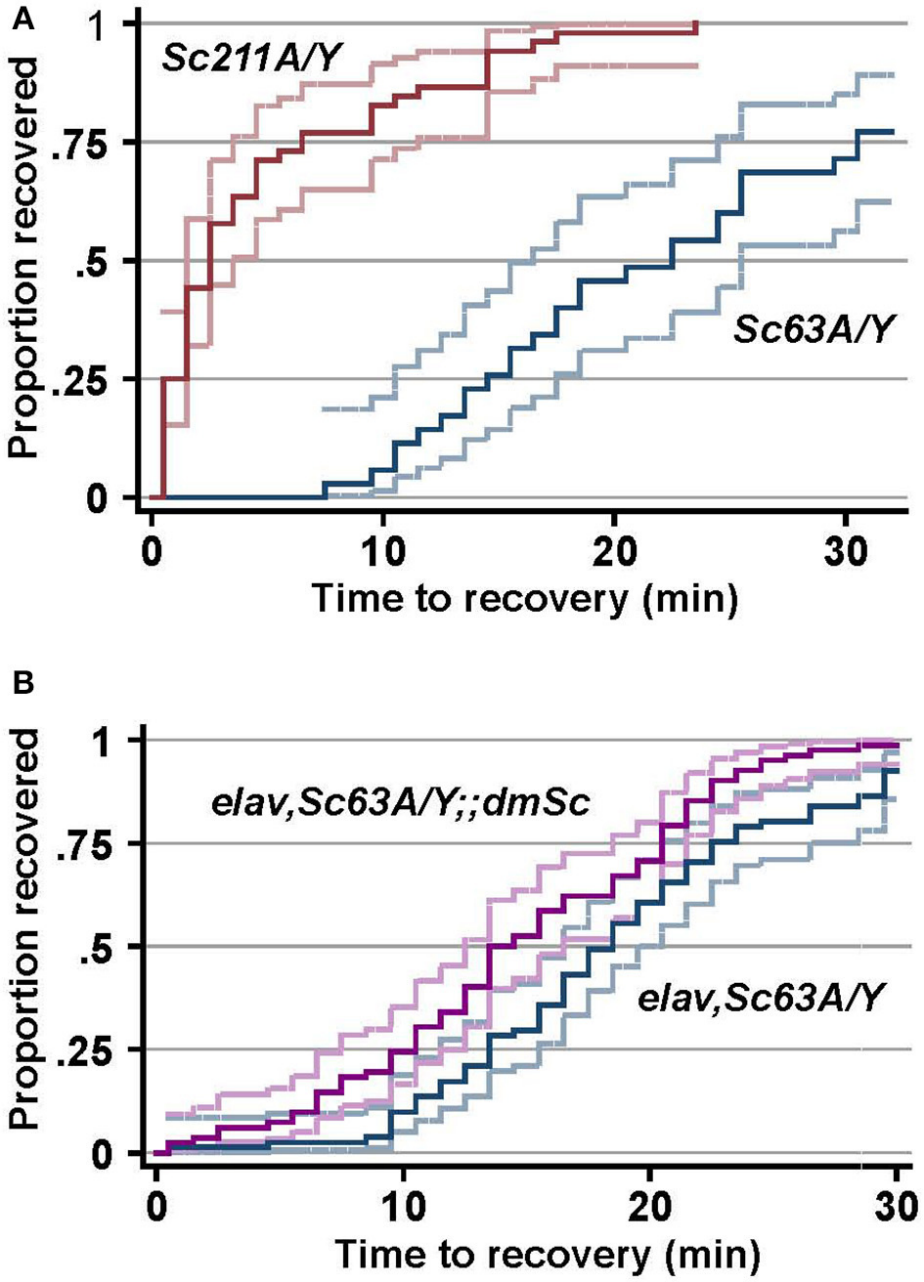
**SCAMP deficiency causes delayed recovery from heat-induced seizures. (A)** The susceptibility to heat-induced seizures of the outcrossed male progeny. **(B)** *elav-GAL4* and *Scamp*^63A^ were genetically combined with *UAS-myc-dmSc* and the susceptibility to heat-induced seizures of the male progeny was determined. The log-rank test for equality of time-to-recovery functions indicated significant difference between the two strains (*p* < 0.001). *N* = 81 or 82 per genotype. Pointwise 95% confidence bands of the estimated survival function are shown by outer lines in pale colors.

Elevated temperatures are a major environmental stress that influences cellular processes including enzymatic activity and the ion flux (Garrity et al., 2010), which affects membrane excitability (Peng et al., 2007) and triggers seizures (Wu et al., 1978; Hoeffer et al., 2003; Wang et al., 2004). Raising the temperature triggers seizures to flies, and the experimentally induced seizures was suggested to mimic some aspects of human epilepsy (Song and Tanouye, 2008). The outcrossed male progeny (*Scamp*^63A^/*Y* and *Scamp*^211A^/*Y*) was subjected to heat-induced seizure susceptibility assay 5–7 days after eclosion. The median recovery time for *Scamp*^211A^/*Y* revertant flies was 2.5 min and 100% of these flies recovered within 23.5 min (*N* = 52). In contrast, the median recovery time of *Scamp*^63A^/*Y* (*N* = 35) was 17.5 min, which is considerably longer than that of revertants (*p* < 0.001 by log-rank test) (Figure 5A). Moreover, 28.6% of *Scamp*^63A^/*Y* flies did not recover even after a 30-min incubation at 22°C (*p* < 0.001 by Pearson's chi^2^-test). Neuronal *UAS-Scamp* restoration experiments showed partial rescue of heat shock recovery. Median recovery time for *elav-GAL4*^*C*155^, *Scamp*^63A^/*Y* flies was 18.5 min (*N* = 81). Inclusion of the *UAS-Scamp* improved the median recovery time to 14 min (*N* = 82), a small but statistically-significant difference (*p* < 0.001 by log-rank test) (Figure 5B). Only 75 out of 81 (92.6%) *elav-GAL4*^*C*155^, *Scamp*^63A^/*Y* flies recovered from heat-shock within 30 min whereas 81 of 82 (98.8%) of the *UAS-Scamp* restored flies did (*p* < 0.05 by Pearson's chi^2^-test).

## Discussion

In the current study, we chose to model *Scamp* deficiency in *Drosophila melanogaster*, one of the most suitable model organisms to test stress-induced behavioral changes. We now show that *Scamp* deficient *Drosophila* mutants exhibit varied behavioral abnormalities including a decline in climbing that is exacerbated by aging, defective odor-associated long-term memory, and increased susceptibility to heat-induced seizures. Rescue of these phenotypes by neuronally-expressed *Scamp* demonstrate that *Scamp* acts within the nervous system. Moreover, restoration of these phenotypes by expression of human SCAMP1 indicates the conserved function for *Scamp*. Rescue of the heat-induced seizure susceptibility by the neuronal *Scamp*-expression was partial. Although the exact mechanism is unknown, it is possible that highly coordinated spatiotemporal *Scamp*-expression may be required to prevent heat-induced seizures, whereas gene expression by the *UAS/GAL4* system is not finely regulated. Aging accumulates intrinsic stress that lead to deterioration in physiological processes including neuronal function. Although there are a number of other possible reasons, neurodegenerative *Drosophila* mutants often experience short lives (Lessing and Bonini, 2009). In this regard, it is interesting to note that *Scamp* nulls showed shorter lifespan than revertant controls. Although the precise mechanism remains to be determined, we postulate that the susceptibility to aging related stress, possibly via neuronal dysfunction, has caused this phenotype. Previous studies reported that SCAMP1-knockout mice were viable and fertile, and mobility of resting animals was not affected (Fernandez-Chacon et al., 1999). Furthermore, depletion of the sole SCAMP gene from the *Caenorhabditis elegans* genome developed no discernable effect on the sensation and spontaneous movement of the worm (Abraham et al., 2006). It is important to note that these studies assessed only movement and sensation of resting animals, and stress- or aging-induced behavioral changes in SCAMP-depleted mice, *Caenorhabditis elegans* or any other model organisms have never been reported. Such stress-induced behavioral changes may indeed reflect integrated brain function. The aging-dependent deterioration of climbing capability was reported to be a characteristic feature associated with *Drosophila* models for Parkinson's disease (Feany and Bender, 2000) and Alzheimer's disease (Iijima et al., 2004; Crowther et al., 2005). It is interesting to note that the behavioral abnormalities we identified in *Scamp* null flies are commonly detected in *Drosophila* models for human neurological disorders, including age-related neurodegenerative diseases and epilepsy.

A predicted *Scamp* hypomorph *w*^1118^*P{EP}EP1593* (the EP transposon insertion 370 bp upstream of the transcription start site) genetically interacts with *ben* (CG32594) in long-term memory (Zhao et al., 2009); however, a role for *Scamp* in memory formation has not been tested. We now show that *Scamp* is necessary for odor-associated long-term memory, phenocopying the *Drosophila Ben* mutants. These results indicate a direct role for neuronal *Scamp* in learning and memory. Intriguingly, pan-neuronal expression of human SCAMP1, but not SCAMP5, partially rescues the long-term memory deficiency phenotype. While SCAMP1 and SCAMP5 show a high degree of similarity in their transmembrane domains, SCAMP5 lacks several putative protein-binding motifs at the N-terminal segment including Asn-Pro-Phe (NPF)-repeats, a Pro-rich domain and a coiled-coil domain. As a consequence, these two isoforms may modulate different biological processes. The N-terminal cytosolic extension was suggested to be crucial for proper targeting for serotonin transporters (Muller et al., 2006) and neuron-enriched Na^+^/H^+^ exchanger NHE5 (Diering et al., 2009). It is possible that the entire structure of *Drosophila* SCAMP including the N-terminal cytosolic extension, which is conserved with mammalian SCAMP1, is needed for learning and long-term memory retention. In contrast to the learning phenotype, we showed that age-dependent climbing impairment of *Scamp* null mutants is rescued by neuronal transgenic expression of both human SCAMP1 and SCAMP5, raising an interesting possibility that this phenotype may be regulated by a common property shared by SCAMP1 and SCAMP5. As both SCAMP1 and SCAMP5 promote Ca^2+^-induced cytokine secretion in rat neuroendocrine model PC12 cells (Liao et al., 2008) and immune cells (Han et al., 2009), these isoforms may exert overlapping functions in certain aspects of secretion. The potential involvement of SCAMP5 in neuronal-activity-dependent endocytosis of synaptic vesicles was also suggested (Zhao et al., 2014) while the involvement of SCAMP1 in endocytosis was also implicated (Fernandez-Chacon and Sudhof, 2000).

Identification and characterization of SCAMP-binding proteins have substantially advanced our understanding on the cellular function of SCAMPs. To better understand the biological role of SCAMPs, extending the cellular level observations to characterization of the molecular network *in vivo* is essential. This can be initiated by generating mutant flies lacking *Scamp* binding protein genes and testing their behavioral trait(s), extending to the characterization of double mutants of *Scamp* and its binding partners. As biologically relevant protein-protein interactions could be weak and transient, isolation of weak interactants particularly integral membrane proteins by biochemical screening methods is a challenge. Our current study has shown the utility of *Drosophila* to address basic questions regarding human *Scamp* function. Future application of the *Scamp* deficient mutants together with the highly sensitive behavioral assays to genome-wide screening may facilitate to unveil a novel neuronal network.

## Concluding remarks

We have established a genetically defined *Drosophila* model and identified quantitative phenotypes, some of which can be rescued by specific human SCAMPs. While the significance of SCAMPs in neuronal functions and their involvement in neurodegeneration have been suggested, *in vivo* evidence supporting the biochemical data has been missing. Our current study has bridged the existing gap in our knowledge and shown that *Drosophila* provides an ideal model to address basic roles of *Drosophila* and human SCAMPs. It will be interesting in the future to extend the genetic interaction studies testing whether mutations to the genes encoding SCAMP-binding proteins confer similar neuronal phenotypes.

### Conflict of interest statement

The authors declare that the research was conducted in the absence of any commercial or financial relationships that could be construed as a potential conflict of interest.

## Supplementary material

Supplementary Figure 1 **Pan-neuronal transgenic expression of myc-tagged *Scamp***. Either *UAS-myc-dmScamp, UAS-myc-hScamp1* or *UAS-myc-hScamp5* and *elav-GAL4* were genetically combined with *Scamp*^63A^ deficiency. Heads were separated and the equal amount of lysates isolated from each line was resolved in SDS-PAGE, transferred to a PVDF membrane and probed with either anti-myc, anti-*Drosophila* SCAMP (dmSc) or anti-Nervana antibody. Nervana is used as a loading control. dmSC (end) = endogenous dmSc.
