# Supplementary figures and images for: Secretory Carrier Membrane Protein (SCAMP) deficiency influences behavior of adult flies
Source: Front Cell Dev Biol. 2014 Nov 18;2:64. doi: 10.3389/fcell.2014.00064 (PMC4235465; doi:10.3389/fcell.2014.00064)

# Supplemental Figure

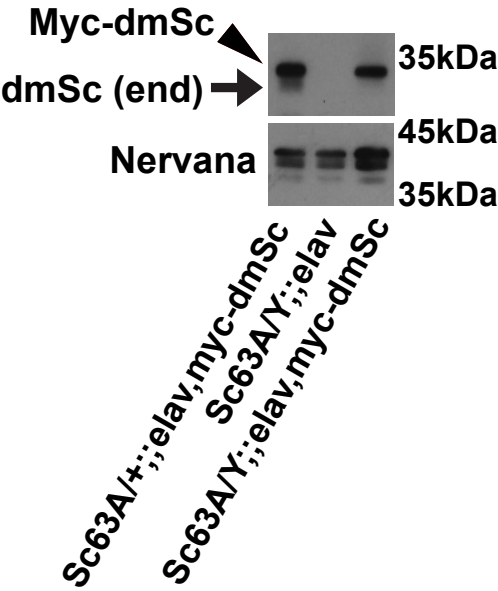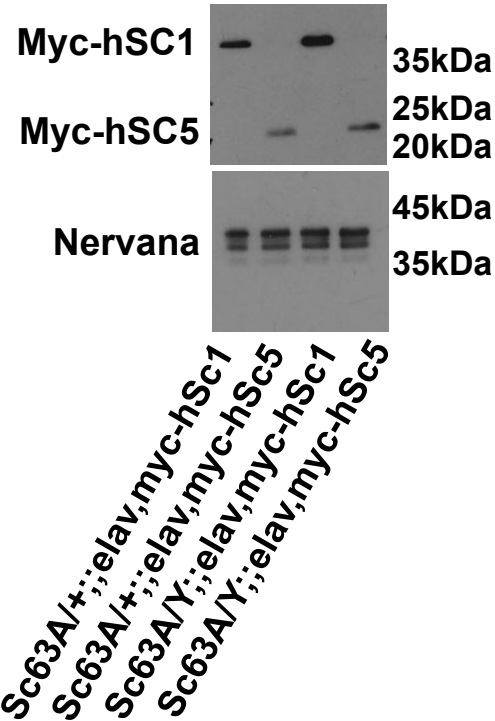

Supplement: Supplementary Figure 1 — Pan-neuronal transgenic expression of myc-tagged Scamp. Either UAS-myc-dmScamp, UAS-myc-hScamp1 or UAS-myc-hScamp5 and elav-GAL4 were genetically combined with Scamp63A deficiency. Heads were separated and the equal amount of lysates isolated from each line was resolved in SDS-PAGE, transferred to a PVDF membrane and probed with either anti-myc, anti-Drosophila SCAMP (dmSc) or anti-Nervana antibody. Nervana is used as a loading control. dmSC (end) = endogenous dmSc. [file Image1.PDF]
